# Continuous sweep versus discrete step protocols for studying effects of wearable robot assistance magnitude

**DOI:** 10.1186/s12984-017-0278-2

**Published:** 2017-07-12

**Authors:** Philippe Malcolm, Denise Martineli Rossi, Christopher Siviy, Sangjun Lee, Brendan Thomas Quinlivan, Martin Grimmer, Conor J. Walsh

**Affiliations:** 1 John A. Paulson School of Engineering and Applied Sciences, Harvard University, Cambridge, MA 02138 USA; 2 Wyss Institute for Biologically Inspired Engineering, Harvard University, Cambridge, MA 02138 USA; 3 Department of Biomechanics and Center for Research in Human Movement Variability, University of Nebraska Omaha, Omaha, NE 68182 USA; 4 University of São Paulo, Ribeirão Preto Medical School, Ribeirão Preto, SP Brazil; 5 Department of Health Sciences and Technology, ETH Zurich, Zurich, 8092 Switzerland

**Keywords:** Exosuit, Protocol, Parameter sweep, Metabolic, Kinematics

## Abstract

**Background:**

Different groups developed wearable robots for walking assistance, but there is still a need for methods to quickly tune actuation parameters for each robot and population or sometimes even for individual users. Protocols where parameters are held constant for multiple minutes have traditionally been used for evaluating responses to parameter changes such as metabolic rate or walking symmetry. However, these discrete protocols are time-consuming. Recently, protocols have been proposed where a parameter is changed in a continuous way. The aim of the present study was to compare effects of continuously varying assistance magnitude with a soft exosuit against discrete step conditions.

**Methods:**

Seven participants walked on a treadmill wearing a soft exosuit that assists plantarflexion and hip flexion. In *Continuous-up,* peak exosuit ankle moment linearly increased from approximately 0 to 38% of biological moment over 10 min. *Continuous-down* was the opposite. In *Discrete*, participants underwent five periods of 5 min with steady peak moment levels distributed over the same range as *Continuous-up* and *Continuous-down*. We calculated metabolic rate for the entire *Continuous-up* and *Continuous-down* conditions and the last 2 min of each *Discrete* force level. We compared kinematics, kinetics and metabolic rate between conditions by curve fitting versus peak moment.

**Results:**

Reduction in metabolic rate compared to *Powered-off* was smaller in *Continuous-up* than in *Continuous-down* at most peak moment levels, due to physiological dynamics causing metabolic measurements in *Continuous-up* and *Continuous-down* to lag behind the values expected during steady-state testing. When evaluating the average slope of metabolic reduction over the entire peak moment range there was no significant difference between *Continuous-down* and *Discrete*. Attempting to correct the lag in metabolics by taking the average of *Continuous-up* and *Continuous-down* removed all significant differences versus *Discrete*. For kinematic and kinetic parameters, there were no differences between all conditions.

**Conclusions:**

The finding that there were no differences in biomechanical parameters between all conditions suggests that biomechanical parameters can be recorded with the shortest protocol condition (i.e. single *Continuous* directions). The shorter time and higher resolution data of continuous sweep protocols hold promise for the future study of human interaction with wearable robots.

**Electronic supplementary material:**

The online version of this article (doi:10.1186/s12984-017-0278-2) contains supplementary material, which is available to authorized users.

## Background

In recent years, there have been a growing number of different academic and industry groups developing wearable robots for walking assistance in able-bodied and clinical populations. Typically, devices have been evaluated and optimized for specific objectives by means of studies where a device parameter is systematically varied while changes in an objective parameter are measured [1–12]. Different studies and devices have targeted different objectives, such as reducing metabolic rate [1–3, 5, 7, 9, 10, 13, 14], increasing walking speed [13], altering muscle activation [6], improving perceived comfort [12] and restoring symmetry [9]. Given that these studies are examining human-machine interactions, it is not surprising that results can vary for different wearable robots [4] or populations [13]. Consequently, there is a need for methods to evaluate the effects of wearable robot parameter settings quickly and efficiently [12, 15, 16] for each new each population and objective and for new types of wearable robots such as soft exosuits [2, 3, 7, 11, 17].

Initially, most experiments with wearable robots used discrete step protocols [1–5, 7, 9, 11]. Discrete step protocols, also called steady-state mapping [15], involve measuring an objective parameter for a certain amount of time at a number of parameter settings within a range of interest. This is followed by a curve-fitting process to identify which parameter setting results in the minimum or maximum objective parameter value. More recently, groups started using continuous sweep protocols [12, 15, 16]. Continuous sweep protocols involve continuously evaluating the goal parameter (e.g. metabolic rate, perception, …) while continuously changing the device parameter by small increments for every subject step or stride.

When focusing specifically on metabolic rate, several challenges arise in the use of discrete step protocols. Due to noise in indirect calorimetry measurements, this type of protocol requires averaging a large number of breaths [18–20] and sometimes even averaging multiple trials or multiple participants to obtain a reliable estimate of the underlying landscape [21, 22]. Due to the delay between changes in local energetic cost and the metabolic response at the pulmonary level, every step of a discrete step protocol must run for 2 to 3 min before steady-state metabolic rate measurements can start [23]. Further, current wearable robots can require several minutes to adapt to the wearer’s gait after a change in controller is applied (e.g. [17]). This also increases the required walking time at each discrete step before the collection of useful data can start. Consequently, to find the optimal parameter settings with a sufficiently high confidence level, participants have to walk for long periods of which the majority is under sub-optimal parameter settings. Walking for a long time with sub-optimal device parameters (e.g. too little or too much assistance) can be challenging for both the robot (e.g. excessive wear and physical drift of the device on the person [24]) and the wearer (e.g. clinical populations may be unable to maintain prolonged effort with suboptimal assistance). Further, high levels of exertion can lead to cardiopulmonary drift [21], decreasing the accuracy of conditions collected late in an experimental session. Thus, required time for data collection, exercise tolerance of the participants and time availability limit the number of parameters settings that can be studied with discrete step protocols. Limited conditions reduce the resolution by which the landscape of metabolic rate versus parameter changes can be analyzed.

In the field of exercise physiology, continuous sweep protocols have been proposed for identifying cardiopulmonary thresholds and have shown promise as a quicker alternative to discrete step protocols which is especially useful for clinical populations that cannot engage in extended activity [19, 21, 23, 25, 26]. Felt et al. [15] suggested using a continuous sweep protocol for optimization, and successfully validated this approach against a discrete step protocol in an experiment involving subject step frequency optimization. In a continuous sweep protocol, metabolic rate at a certain parameter setting is delayed compared to a discrete step protocol. Felt et al. developed a method that removes this delay by modeling changes in metabolic rate with a first-order linear dynamic model with a single time constant based on work by Selinger et al. [22]. Having been successful used in exercise physiology and studying step frequency, it will be important to understand if continuous protocols can be applied to parameter tuning for wearable robots, in particular given the known complexity of human-exoskeleton interactions [27].

The primary focus of the present study was to evaluate differences in metabolic rate and gait biomechanics between evaluation protocols that use continuous and discrete variations of actuation parameters. In a continuous sweep protocol, it is known that there is an apparent lag or delay between parameter changes and the corresponding changes in metabolic rate. This can be due to the bulk effect of physiological dynamics and biomechanical response delay. The apparent delay in metabolic rate is not an absolute delay but is known to follow an exponential evolution over time. Conversely, we define the time that is required by a trained user to change biomechanics in response to a parameter change as “biomechanical response delay”.

Our specific aims and hypotheses are as follows: (1) Measure if there is biomechanical response delay in continuous conditions by comparing biomechanical metrics between continuous and discrete conditions. We hypothesize that potential biomechanical response delay is small or non-existent when the continuous changes in wearable robot parameters are gradual and thus would be easy for participants to adapt to, except if the parameter change is such that the user can never adapt to it, such as if the force is too high to allow walking. (2) Evaluate the physiological dynamics during the continuous conditions by comparing reduction in metabolic rate between continuous and discrete conditions. We hypothesize that measured reductions in metabolic rate in continuous conditions will be lagging behind the values that would be expected during steady-state testing. In turn we expect this will result in higher reductions measured in the continuous condition where the peak force goes from high to low, and lower reductions in the continuous condition where the peak force goes from low to high and the reductions in discrete conditions being halfway between both continuous condition directions. (3) Evaluate two methods to correct for physiological dynamics during testing with a continuous protocol: taking the average of two sweep directions of a continuous condition and using the instantaneous metabolic cost estimation from Selinger et al. [22] and Felt et al. [15]. Building further on our earlier hypothesis that metabolic reduction in a discrete sweep is midway between continuous sweep directions we hypothesize that taking the mean of both directions of a continuous sweep will closely estimate the results of a discrete sweep. Also, building further on our hypothesis that biomechanical response delay will be small or non-existent we hypothesize that the instantaneous cost estimation using an assumed fixed time constant similar to previous literature will closely estimate metabolic reduction from discrete sweep.

To validate the use of continuous and discrete protocols for optimizing wearable robotics we compared both protocols in a parameter study with a soft textile based exoskeleton, called exosuit, developed by our group [2, 3, 7, 11, 17]. In previous studies with other wearable robots (e.g. [5, 28]), groups have found that higher assistance magnitude can lead to higher reductions in metabolic rate while other studies in have found that higher assistance magnitude can also lead to less reduction in metabolic rate (e.g. [5]). Therefore, we believe it is relevant to do an experiment involving changing the assistance magnitude to compare both parameter study methods for measuring the effect of peak assistance force or moment with our soft exosuit.

## Methods

### Participants

We tested seven participants (27 ± 5 yrs.; 68 ± 10 kg; 1.7 ± 0.1 m; mean ± SD). The study was approved by the Harvard Longwood Medical Area Institutional Review Board and all experiments were conducted in accordance with this approved protocol. All participants provided written informed consent.

### Conditions

Participants walked on a treadmill at 1.5 m s^−1^ under two conditions while parameters were varied: A *Discrete* step condition and two *Continuous* sweep conditions (*Continuous-up* and *Continuous-down*).

In *Discrete*, participants underwent a series of 5-min periods with steady peak forces at zero (*Powered-off*), 18.7 (*Low*), 37.5 (*Med*.), 56.2 (*High*), and 75.0% body weight (*Max*.). This is similar to how the majority of standard parameter studies were conducted in the literature [1–5, 7, 10, 11].

In *Continuous-up*, the desired peak exosuit ankle force increased from zero to 75% body weight over 10 min. *Continuous-down* was the opposite. Before each *Continuous* condition participants walked for 4 min under the assistance level from the start of the parameter sweep and after each *Continuous* condition they walked for another 3 min under the assistance level from the end of the parameter sweep. Assuming a 42-s time constant [22] and knowing that most of the change in exoskeleton force happens quickly just after starting the exoskeleton controller, this should allow sufficient time for metabolic rate to approach the steady state value.

### Protocol

Before the testing session participants did a training session that involved a total of one-hour walking with all the conditions that would be repeated during the testing session.

During the testing session, participants began with an 8-min warm-up period during which they briefly experienced all force magnitudes before the actual measurements began. We randomized the order of the conditions such that participants either started by completing all the *Continuous* conditions or by completing all of the force level of the *Discrete* condition. Within the *Continuous* conditions participants alternatively started with either *Continuous-up* or *Continuous-down*. In *Discrete*, we also randomized the order of the five force levels and grouped them into two uninterrupted walking blocks, each 15 min in length and each containing a *Powered-off* trial for relative comparison of metabolic data. Between all testing blocks we gave a 5-min break.

### Exosuit

**Fig. 1:**
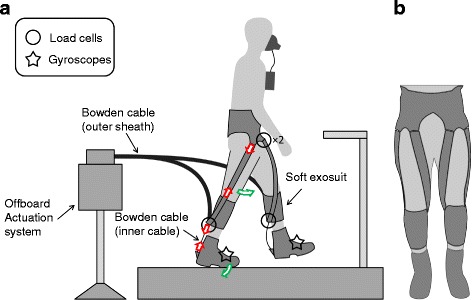
Experimental setup. **a** Participant wearing a soft exosuit, the actuation system (offboard, Bowden cable, sensor locations). Part of this figure is reproduced from [30]. **b** Frontal view showing the medial and lateral straps running from the front of the waist-belt to the rear of the calf-wraps

The participants wore a soft exosuit consisting of a spandex base layer, a waist belt, a calf wrap on each leg, and two vertical straps per leg crossing from the back of the calf wrap, through the center of the knee joint axis, to the front of the waist belt (Fig. 1). This configuration assists both ankle plantarflexion and hip flexion [11, 17].

### Actuation system and sensors

We used an offboard actuation system to generate assistive forces and Bowden cables to transmit the forces to the exosuit at the ankle joint. The Bowden cable sheaths were connected to the back of the calf wrap and the inner cables were connected to a metal bracket at the back of the heel of the boot. As the motor retracted the cable, the distance between the two attachment points was shortened, generating a tensile force that was distributed between the calf wrap and the vertical straps, resulting in a plantarflexion moment around the ankle and flexion moment around the hip [11, 17].

We attached one gyroscope (LY3100ALH, STMicroelectronics, Geneva, Switzerland) on each foot to segment gait cycles and attached one load cell (LTH300, FUTEK Advanced Sensor Technology, Irvine, CA, USA) on each calf wrap to measure the force exerted at the Bowden cable attachment. To measure the assistive force transmitted to the hip joint, we attached two load cells (LSB200, FUTEK Advanced Sensor Technology, Irvine, CA, USA) in series with the two vertical straps and the waist belt.

### Control

We used a triangular motor position profile to deliver assistive moments similar to the biological ankle moment pattern considering both ankle joint kinematics and human-suit effective stiffness, as described in [11]. During each stride, the motor retracted the Bowden cable at a constant rate starting from the heel-strike until the end of the stance phase. Just before the swing phase, the motor released the cable out such that the system did not hinder the wearer’s natural motion during the swing phase. The system performed a force-based position control on a stride-by-stride basis similar to [7], which made decisions on the slope of the triangular trajectory for the next stride by comparing the desired and the actual peak forces at the end of each stride. The desired peak force for each stride was varied depending on the experimental conditions. In the *Discrete* conditions, the desired peak force was set as a constant at each desired force level. On the other hand, in *Continuous-up/down* conditions, the desired peak force was set to linearly increase (*Continuous-up*) or decrease (*Continuous-down*), as described in the experimental protocol section. In *Discrete* the controller required about 1 min to reach the desired assistance magnitude since the controller was programmed to gradually adjusted the motor position profile over a number of steps. In *Continues-up* and *Continuous-down* the controller followed the desired assistance magnitude with a delay of about 3 s. The controller achieved a mean error in peak force of 0.027 ± 0.085 N kg^−1^ and a mean absolute error in peak force of 0.308 ± 0.049 N kg^−1^.

### Measurements

We calculated metabolic rate based on rates of oxygen consumption and carbon dioxide production [29] measured via indirect calorimetry (K4b2, Cosmed, Rome, Italy). We analyzed metabolic rate of every participant of *Continuous-up* and *Continuous-down* and the last 2 min of each of the force levels of *Discrete.* We did not use noise eliminating methods other than averaging these last 2 min. We calculated the change in metabolic rate between each active condition and *Powered-off*. For *Discrete* we used the *Powered-off* that we recorded within the same 15-min bout of walking. For *Continuous-up* we used the last minute at *Powered-off* before the start of *Continuous-up* and for *Continuous-down* we used the last minute at *Powered-off* after the end of *Continuous-down*.

We measured body segment motions using a motion capture system (Vicon, Oxford Metrics, Oxford, UK; 120 Hz). We collected three-dimensional ground reaction forces using an instrumented split-belt treadmill (Bertec, Columbus, OH, USA; 2160 Hz). We filtered all marker and force signals using a zero-lag fourth order low pass Butterworth filter with a 5–9 Hz cut-off frequency that we selected using a custom residual analysis algorithm (MATLAB, MathWorks, Natick, MA, USA). We calculated sagittal plane moment arms of the exosuit at the ankle and hip, joint angles, joint moments, and joint powers for the right leg using Visual 3D (C-Motion, Rockville, MD, USA). To evaluate biomechanical response delay, we analyzed peak joint angles that were previously shown to significantly change with increasing peak exosuit ankle moment in [30]. To have a measure of global biomechanical adaptation with same units as metabolic rate (i.e. W kg^−1^), we also calculated a center-of-mass based metabolic rate estimate [4, 5, 31] from the sum of all positive and negative center-of-mass power, minus exosuit power, multiplied by respective muscle efficiencies from Margaria et al. [32].

We used an automatic gait event detection algorithm (Visual3D, C-Motion, Rockville, MD, USA) to determine heel strikes and segment kinematic and kinetic data into gait cycles. From *Continuous-up* and *Continuous-down* we processed the entire 10 min of biomechanical data and from *Discrete* we processed the last minute of each force level.

### Data analysis

*Continuous-up* and *Continuous-down* each contain a few hundred data points (metabolic rate for every breath and biomechanics for every stride) whereas *Discrete* contains five data points (averaged data from five force levels). To allow comparison between both *Continuous-up* and *Continuous-down* with *Discrete*, we had to reorganize the data prior to running statistics due to this unequal sampling rate.

**Fig. 2:**
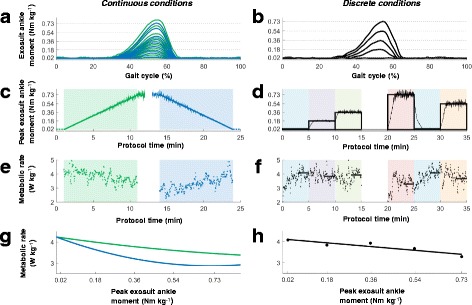
Data organization in *Continuous-up*, *Continuous-down* and *Discrete* based on example data from one participant**. a** Peak exosuit ankle moment over the gait cycle of *Continuous-up* and *Continuous-down* condition. The peak moment values ranged from *Powered-off* to *Maximum* (*Max.*) applied moment (*Continuous-up*) and from *Max.* to *Powered-off* (*Continuous-down*). **b** Peak exosuit ankle moment over the gait cycle of *Discrete*. Peak moment values at *Powered-off*, *Low*, *Med.*, *High,* and *Max.* applied moment at the ankle. **c** Peak moment values and time for *Continuous-up* (*green line*) and *Continuous-down* (*blue line*). The *thin line* shows actual peak moments and the *thick line* shows curve fit of moment versus time. The order of *Continuous-up* and *Continuous-down* was randomized for each participant. **d** Five minutes at each moment level in *Discrete* in a randomized order. The *thin line* shows actual peak moments and the *thick line* shows commanded peak moment. **e** Metabolic rate over time for *Continuous-up* (*blue points*) and *Continuous-down* (*green points*). Points represent breaths. **f** Metabolic rate over time of each *Discrete* moment level. **g** Metabolic rate fitted at each peak moments level from *Powered-off* to *Max.* **h** Average metabolic rate of the last 2 min at each moment level from *Powered-off* to *Max*

**Fig. 3:**
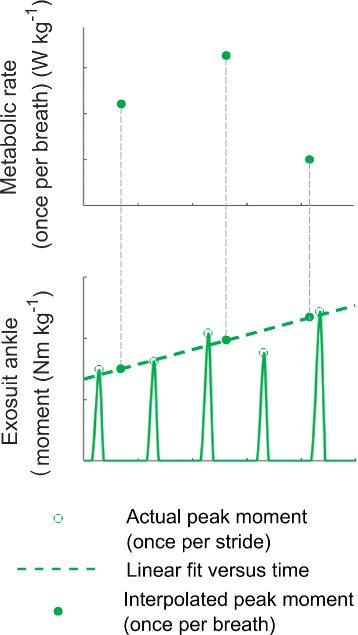
Exosuit ankle moment interpolation. Metabolic rate is sampled once per breath. Exosuit ankle peak moment is sampled once every stride. In order to be able to relate metabolic rate to peak moment we calculated a linear fit between peak exosuit ankle moment and experiment time. This linear fit is then evaluated at time points corresponding to metabolic measurements

According to the design of *Continuous-up* and *Continuous-down*, stride-by-stride peak exosuit ankle moment varied linearly with time (Fig. 2a, c). Because metabolic rate measurements took place breath by breath, this did not match up temporally with any given stride’s peak exosuit ankle moment. To accommodate this, we first calculated a linear fit between peak exosuit ankle moment and experiment time. Evaluating this fit at the time points corresponding to metabolic measurements provided a corresponding peak exosuit ankle moment for every metabolic rate measurement in *Continuous-up* and *Continuous-down* (Fig. 3).

Next, in the *Continuous-up* and *Continuous-down*, we fit breath-by-breath metabolic rate to peak exosuit ankle moment with a second order polynomial (Fig. 2g). In *Discrete*, we fit the steady-state metabolic rate to peak exosuit ankle moment with a second order polynomial (Fig. 2h). The selection of the curve fitting order was based on evaluation of significances of coefficients trying different options from third to first order (Additional file 1). We used the same methods for fitting stride-by-stride biomechanical parameters versus peak exosuit ankle moment.

**Fig. 4:**
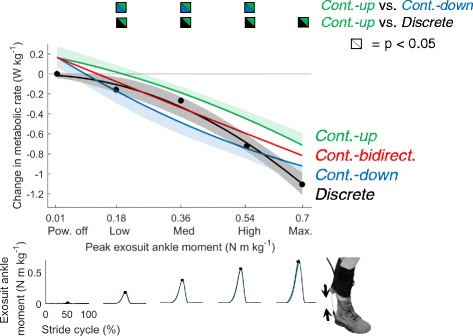
Change in metabolic rate plotted against exosuit ankle peak moment. *Black*, *green*, *blue* and *red line* respectively represent population average second order polynomial curve fits for *Discrete*, *Continuous-up*, *Continuous-down* and the average of *Continuous-up* and *Continuous-down* called *Continuous-bidirectional*. *Shaded* areas represent standard error of *Discrete*, *Continuous-up* and *Continuous-down* (for *Continuous-up* standard error is only plotted in the positive direction and for *Continuous-down* it is only plotted in the negative direction). *Bi-colored symbols* represent significant differences (*P* ≤ 0.05) between the curve fits of the different conditions with the colors in the symbol evaluated at peak moment levels from *Discrete*. Time series plots show population average exosuit ankle moment profiles at the peak moment levels of *Discrete*

**Fig. 5:**
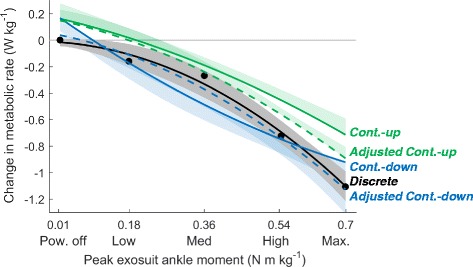
Instantaneous metabolic rate estimation. *Adjusted Continuous-up* and *Adjusted Continuous-down* based on instantaneous metabolic rate estimation [15, 22] assuming a fixed time constant of 42 s based on [22]. *Dashed green* and *blue line* represent population average for instantaneous metabolic rate estimation respectively for *Continuous-up* and *Continuous-down*. *Black line* represents population average for second order polynomial curve fit from *Discrete*. *Bi-colored squares* represent significant differences

We evaluated two ways of compensating for physiological dynamics in the data from *Continuous-up* and *Continuous-down*. As a first method, we performed our curve fitting on the combined data from *Continuous-up* and *Continuous-down*, a method we define as *Continuous-bidirectional* (Fig. 4). As an alternative approach to adjust for physiological dynamics, we also utilized the instantaneous cost mapping function outlined by Felt et al. [15] and Selinger et al. [22] (Fig. 5). We applied their methods with a fixed assumed time constant of 42 s (this value was chosen based on the average observed time constant in [22]) on the section of the data including the last minute of steady-state data before the continuous parameter change and the 3 min of steady state data after the continuous parameter change. The results of the instantaneous cost mapping of *Continuous-up* and *Continuous-down* were respectively called *Adjusted Continuous-up* and *Adjusted Continuous-down*. For all conditions (*Discrete*, *Continuous-up, Continuous-down, Adjusted Continuous-up* and *Adjusted Continuous-down*) we calculated all the curve fits on individual data and then averaged to plot the population average. In addition to applying the instantaneous cost mapping with a fixed assumed time constant of 42 s we also solved for the actual subject time constants that optimally reduced the sum of the squared differences between *Continuous-up* and *Continuous-down*, between *Continuous-up* and *Discrete* and between *Continuous-down* and *Discrete* and we reported these average best fitting time constants.

### Statistics

For testing differences between conditions, we first evaluated the individual curve fits of each condition at the different peak exosuit ankle moment levels of *Discrete*. We conducted repeated-measures ANOVA to test for significant differences between *Continuous-up*, *Continuous-down* and *Discrete* for the curve fits evaluated at the different peak moment levels in order to evaluate absolute differences between conditions. We also used repeated-measures ANOVA to check for differences between conditions for the delta between the curve fits evaluated at the highest peak moment minus the curve fits evaluated at the lowest peak moment in order to evaluate the effects of different conditions on the relative parameter changes over the entire peak moment range. *Continuous-bidirectional* was included in this analysis as a fourth condition. We applied these aforementioned analyses on metabolic rate, peak joint angles and the center-of-mass based metabolic rate estimate.

We also separately applied the same analysis to test for significant differences between *Adjusted Continuous-up, Adjusted Continuous-down* and *Discrete*. On measures that showed significant condition effects, we performed paired t-tests to compare conditions. We then compared *Continuous-up* versus *Continuous-down* to assess if differences existed that may be due to metabolic or biomechanical response delay. We also compared *Continuous-bidirectional, Continuous-up* and *Continuous-down* each versus *Discrete* to understand which condition may be a best match to *Discrete*. All statistical analyses were within-subject so this does not take into account between-subject variability. We used a significance threshold of *α* = 0.05.

## Results

### Exosuit kinetics

Peak exosuit ankle moments in *Discrete* were 0.014 ± 0.002, 0.179 ± 0.004, 0.360 ± 0.060, 0.544 ± 0.010, 0.699 ± 0.0170 N m kg^−1^ (mean ± s.e.m.) respectively for the *Powered-off*, *Low*, *Med.*, *High* and *Max*. Peak exosuit ankle moments during the minute before and after *Continuous-up* were 0.015 ± 0.002 and 0.686 ± 0.035 N m kg^−1^. Peak exosuit ankle moments during the minute before and after *Continuous-down* were 0.652 ± 0.034 and 0.013 ± 0.002 N m kg^−1^. In summary, these metrics show that we were able to sweep peak exosuit ankle moments over similar ranges in all conditions.

### Biomechanical adaptation

There were no significant differences between any of the methods in joint angles and the center-of-mass based metabolic rate estimate (all *p*-values >0.24) (Table 1)*.* However, the center-of-mass based metabolic rate estimate showed strong significant correlations with the actual change in metabolic rate (*P* < 0.001 and *R* ^2^ = 0.70, Pearson’s correlation test). In summary, these results show that there were no significant differences in the evaluated biomechanical parameters between all conditions.

**Table 1:**
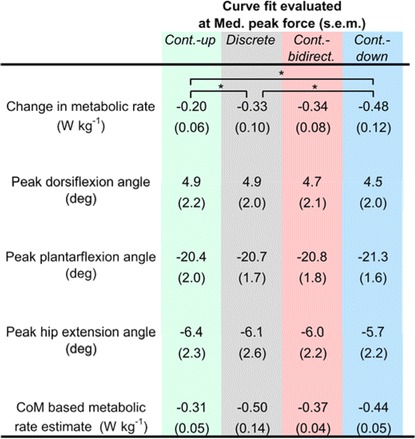
Biomechanical parameter comparison

Individual curve fits of biomechanical parameters and metabolic rate were evaluated at the peak exosuit moment of *Med.* for *Continuous-up*, *Discrete*, *Continuous-bidirectional* and *Continuous-down* Brackets indicate significant differences (*P* ≤ 0.05). The peak joint angles in the table were selected based on the kinematical parameters that were shown to significantly increase or decrease with increasing peak exosuit ankle moment in [30]. Center-of-mass based metabolic rate estimate was selected as a global parameter that would represent the entire biomechanical changes and would have a good correlation with metabolic rate in a magnitude variation experiment [5, 28]. For comparison, change in metabolic rate is also reported in the table

### Metabolic rate

Metabolic rate followed a monotonically decreasing trend versus peak exosuit ankle moment in all the conditions (Fig. 4). Change in metabolic rate in *Discrete* varied from zero at *Powered-off* to −1.11 ± 0.14 W kg^−1^ at *Max*. Change in metabolic rate in *Continuous-up* varied from +0.17 ± 0.12 W kg^−1^ at the start to −0.70 ± 0.11 W kg^−1^ at the end. Change in metabolic rate in *Continuous-down* ranged from −0.95 ± 0.14 W kg^−1^ at the start to +0.15 ± 0.10 W kg^−1^ at the end. Change in metabolic rate in *Continuous-bidirectional* ranged from +0.16 ± 0.11 W kg^−1^ at *Powered-off* to −0.83 ± 0.13 W kg^−1^ at *Max*.

The curve fit of *Continuous-up* had a significant positive intercept (*P* = 0.02). The curve fit of *Continuous-down* also had a large positive offset trending toward significance (*P* = 0.06). Reduction in metabolic rate in *Continuous-up* was significantly smaller than in *Discrete* at all peak exosuit moment levels from *Low* to *Max.* (*P* = 0.01, 0.03, 0.007, 0.03). ()The average slope of the reduction in metabolic rate over the tested peak moment range was significantly smaller in *Continuous-up* than in *Discrete* (*P* = 0.02). In *Continuous-down* and *Continuous-bidirectional* the average slopes of the reduction in metabolic rate over the tested peak moment range were not different from *Discrete* (*P* = 0.98 and 0.33 respectively). In summary, the results show that there were large significant differences in metabolic rate at the different peak moment levels between conditions but the average slope of the trend was more similar between conditions.

After applying the instantaneous metabolic rate estimation with a fixed time constant of 42 s [22], *Adjusted Continuous-up* was still higher than *Adjusted Continuous-down* at *High* (*P* = 0.02) which indicates that the time constant was larger than we expected. *Adjusted Continuous-up* was higher than *Discrete* at *Powered-off* and *High* (*P* = 0.04 and 0.02). There was also a trend toward a significant difference between the instantaneous metabolic rate estimate from *Adjusted Continuous-up* and *Discrete* at *Max.* (*P* = 0.06). This indicates that even after applying the instantaneous cost estimation *Continuous-up* and *Continuous-down* did not closely match with *Discrete*. When we solved for the actual subject time constants we found that a time constant of 106 +/−29 s optimally reduces the sum of the squared differences between *Continuous-up* and *Discrete* and a time constant of 49 +/− 20 optimally reduces the sum of the squared differences between *Continuous-down* and *Discrete*. Additional file 2 shows a simulation of what the difference between conditions would have looked like if differences would have been exclusively due to physiological dynamics following an exponential change with a time constant of 42 s [22]. In summary, these results show time constants that are higher than the assumed time constant of 42 s and that vary depending on which conditions are tried to match.

## Discussion

Understanding how to select parameter values for wearable robots is important for guiding their development. This study examines the differences in biomechanical and physiological outcomes while varying parameter values in a continuous (*Continuous-up* and *Continuous-down*) and discrete step (*Discrete*) manner. We varied the level of exosuit assistance delivered at the ankle joint through a multi-articular soft exosuit and compared biomechanical and metabolic measurements to those collected at steady state conditions at four different discrete assistance levels. In addition, we expected differences between the *Continuous-up* and *Continuous-down* conditions due to physiological dynamics and potential biomechanical response delay.

For the selected kinematic and kinetic parameters that we evaluated, we found no differences between *Continuous-up* and *Discrete* and between *Continuous-down* and *Discrete*. Further, we did not find any difference in variability parameters between the *Continuous-up* and *Discrete* and between *Continuous-down* and *Discrete* (Additional file 3). This indicates that participants had no difficulty in adapting to the slow linear increase or decrease in peak exosuit ankle moment in the *Continuous-up* and *Continuous-down* conditions. This echoes the interpretation of a recent treadmill walking study in which the authors assumed that participants can perfectly adapt to treadmill belt speed changes if they are implemented in a slow linear way similar to *Continuous-up* and *Continuous-down* conditions [33]. To check how important the slow linear increase or decrease in peak exosuit ankle moment in *Continuous-up* and *Continuous-down* was to avoid biomechanical response delay we did an additional analysis to check if there was response delay in biomechanics in the *Discrete* conditions (Additional file 4). Based on average results it appears that there was no biomechanical response delay even in the changes between the *Discrete* force levels. It should be noted that in even the *Discrete* conditions the changes between force levels were slow due to the limitations of the soft exosuit controller so it is possible that biomechanical response delay would have existed with more abrupt transitions between the *Discrete* force levels.

Since there were no differences between *Continuous-up* and *Discrete* and between *Continuous-down* and *Discrete*, our results indicate that for kinematic or kinetic measurements *Discrete* could be replaced by either only *Continuous-up* or only *Continuous-down* which required 4 + 10 min for data collection whereas *Discrete* required 30 min). It should be kept in mind however that in addition to the actual protocol time participants also completed a training day and warm-up that constituted an additional hour and 15 min of walking and that the results could also have been different if participants had different levels of training.

Another potential advantage of replacing discrete protocols with continuous protocols is that the latter give better resolution of parameter setting responses, however, it is not clear if this outweighs the accuracy associated with collecting minutes of data at a single parameter setting. Further studies are required to fully understand this tradeoff. Evaluating the effects of parameter settings between the actual steps of a discrete protocol requires interpolation, whereas with a continuous sweep a higher resolution of parameter settings within the sweep range are actually tested.

When we look at the absolute reductions in metabolic rate, as expected, reduction in metabolic rate was smaller in *Continuous-up* than in *Continuous-down* at most peak moment levels (Fig. 4). This finding is consistent with the knowledge that metabolic rate follows an exponential change over time, characterized in earlier studies comparing continuous and discrete step frequency changes [15, 22], along with exercise physiology studies [23, 34].

When we evaluated the average slope of metabolic reduction over the range in peak moment, we found that the slope in *Continuous-up* was significantly smaller than in *Discrete* but there was no significant difference between *Continuous-down* and *Discrete*. A possible explanation for the closer match between *Continuous-down* and *Discrete* can be found in the simulation prediction for the effect of physiological dynamics on *Continuous-up* and *Continuous-down*. In *Discrete* the slope of metabolic rate versus change in peak moment was steeper around the highest peak moment levels. As a result of this, the effect of the physiological dynamics can be expected to be larger at higher peak moments (Additional file 2). Since metabolic rate is at steady-state at the very start of *Continuous-down* this is expected to favorably minimize the error due to the physiological dynamics when the peak moment is still close to *Max.* (but shortly after that the error due to physiological dynamics will re-appear). In our study, we found that *Continuous-down* by itself could potentially replace *Discrete* for measuring the relative changes in metabolic rate which would represent a time-saving of about 50% compared to *Discrete*. In protocols with other devices and evaluation parameters it can be hypothesized that a continuous sweep protocol that starts from the steepest side of the metabolic landscape may be the best uni-directional sweep candidate for replacing a discrete protocol.

To understand if we could completely correct for the physiological dynamics, we explored two different approaches that have been previously described in the literature. A first method was suggested by Felt et al., 2015 and consists in taking the average of measurements obtained from increasing and decreasing parameter sweeps. We applied this method by calculating *Continuous-bidirectional,* based on curve fitting on the combined data from *Continuous-up* and *Continuous-down*. We found that there were no significant differences between *Continuous-bidirectional* and *Discrete* both in the relative slope of metabolic reduction over the range in peak moment and the absolute metabolic reduction at all force levels (Fig. 4). There were also no significant differences in reduction in metabolic rate at the different force levels between *Continuous-bidirectional* and *Discrete* (Fig. 4). In our protocol, *Continuous-bidirectional* required about the same time as *Discrete* (respectively 28 and 30 min).

A second approach to account for the physiological dynamics is the instantaneous metabolic rate estimation from Selinger et al. [22]. They showed that the change in metabolic rate after a parameter change can be fitted with a first-order differential equation. In an experiment involving step frequency optimization Felt et al. [15] proposed to use this method to find the best fitting polynomial of instantaneous metabolic rate from only one continuous sweep direction and showed that it can achieve similar accuracy for optimization as a discrete step protocol at only 1/6th of the time. In our data, applying the instantaneous metabolic rate estimation using a fixed time constant of 42 s eliminated the significant differences between all conditions. When solving for the individual time constants that reduce the response delay between *Continuous-up* and *Discrete* and between *Continuous-down* and *Discrete* we found time-constants that are much larger than the previously assumed time constant of 42 s. We also found that the best fitting time constants that explain the difference between *Continuous-up* and *Discrete* are higher than the best fitting time constants that explain the difference between *Continuous-down* and *Discrete*. This is different from what has been found when comparing on-off transitions in exercise physiology [35].

These conclusions can be compared to recent relevant results from Koller et al. [16]. They compared a bidirectional continuous sweep protocol and a discrete step protocol for optimizing actuation timing of an ankle exoskeleton. In their study, they evaluated the standard deviation of the probability distribution of the estimated optimum actuation timing from both conditions. They found a marginally higher variability in the distribution of the optimum in their continuous sweep condition than in their discrete step condition and attributed this to a much shorter time that was used in their continuous sweep protocol. If we had found a local minimum in metabolic rate in all conditions, we could have compared the validity and repeatability of both methods, similar to Koller et al. [16]. For reference the confidence interval analysis that was used in Koller et al. is shown in Additional file 5.

The positive offset in metabolic rate as a function of peak exosuit ankle moment in *Continuous-up* is an unexpected result. This results deviates from the hypothesis that the metabolic reduction in *Discrete* would be midway between the metabolic reduction in *Continuous-up* and *Continuous-down* (Fig. 5b) [15]. There are different possible explanations for the significant positive offset in change in metabolic reduction in *Continuous-up*. One possible reason could be under-fitting. Raw breath-by-breath values from three of the seven participants show a small increase in metabolic rate between *Powered-off* and *Low* before metabolic rate descends toward *Max*. This increase in metabolic rate between *Powered-off* and *Low* could be noise or it could indicate that the 4-min walking period before the continuous increase in peak exosuit moment started was insufficient to reach metabolic steady-state. In *Discrete*, *Powered-off* is one of five fitting points (the other four being *Low* to *Max.*) however, *Powered-off* is not part of the curve fitted data in *Continuous-up*, *Continuous-down* or *Continuous-bidirectional*. As such, the curve fits in the continuous conditions will be less likely to go through zero metabolic rate at *Powered-off*.

A limitation of all comparisons of *Continuous-up*, *Continuous-down* and *Continuous-bidirectional* versus *Discrete* is that *Discrete* is also affected by normal noise and variability in metabolic rate and wearer adaptation. It should also be noted that our findings from comparing different protocols cannot be taken as general guidelines, since the results dependent on the protocol modalities [23, 25]. Longer continuous sweep conditions could give better data resolution, but would increase the risk of slow metabolic drift [21] confounding the trend. Shorter continuous sweep conditions would reduce the risk for metabolic drift but would reduce the data resolution and might be more challenging for participants to adapt to and this could possibly introduce biomechanical response delay. Results could have been affected by the time to reach steady state in *Discrete* as well as in the steady-state periods before *Continuous-up* and *Continuous-down*. While the time that we allowed should have been sufficient to approach the steady-state value assuming a time constant of 42 s the fact that we found higher time values when analyzing the individual time constants indicates that this could have affected our results. Results could also have been different if participants were given a shorter or longer training and warm-up time. With shorter training the relative importance of small time savings would be larger but it could also be possible that participants would be less able to quickly adapt to the continuous conditions given less training beforehand. In future studies with discrete and continuous sweeps it could be interesting collect data from the training day to investigate if there is more biomechanical response delay when participants first experience changes in exosuit assistance. While we did not find evidence of biomechanical response delay in kinematics and total body kinetics it could be possible that there would changes in muscle activation (e.g. co-contraction) that could cause and explain changes in metabolic cost. However, in this study we did not measure EMG data and so future studies would be required to investigate if there was a link between changes in muscle activation and metabolic rate. For the *Adjusted Continuous-up* and *Adjusted Continuous-down* calculations it could also be interesting to rigorously calculate and use the individual time constant based on extra tests with a large number of small discrete parameter changes. Other limitations are the number of participants that were tested and the fact that we did not use correction for multiple testing. Because of all these limitations the results of this study should be rather interpreted as an explorative analysis of the possible advantages and disadvantages of discrete and continuous protocols rather than a recommendation for either of them over the other. Finally, it should also be noted that aside from continuous protocols other protocols could provide improvements and are being studied such as gradient based methods [14, 15].

## Conclusion

Different groups successfully developed wearable robots for walking assistance, but there is still a need for methods to quickly tune actuation parameters for each robot and population. Discrete step protocols have traditionally been used for evaluating responses to parameter changes, but these are time-consuming. Recently, protocols have been proposed where a device parameter is changed in a continuous way. In the present study, we show that by continuously varying assistance magnitude (either increasing, *Continuous-up*; or decreasing, *Continuous-down*) with a soft exosuit, we can achieve a shorter time and higher resolution data collection for specific applications compared to a protocol with discrete step conditions (*Discrete*). As expected, reduction in metabolic rate was smaller in *Continuous-up* than in *Continuous-down* at most peak moment levels due to the physiological dynamics associated with metabolic measurements. When evaluating the average slope of metabolic reduction over the entire peak moment range there was no significant difference between *Continuous-down* and *Discrete*. Thus, a unidirectional continuous sweep, starting from the steepest side of the landscape of metabolic rate versus parameter change, may replace a discrete step protocol for evaluating the average slope of metabolic rate over peak moment. Attempting to correct for the physiological dynamics by taking the average of *Continuous-up* and *Continuous-down* successfully removed all significant differences versus *Discrete*. However, this result should not be interpreted as an argument against the use of the adjustment methods from Felt et al. [15] and Selinger et al. [22]., On the contrary, our results also showed that the *Adjusted Continuous-up* and *Adjusted Continuous-down* correctly estimated *Discrete*.

For all biomechanical parameters, we found that *Continuous-up* and *Continuous-down* both closely matched with *Discrete* and therefore could replace *Discrete*. Such a unidirectional sweep would save approximately half the time of a discrete step protocol for the methods used in this study. Another potential advantage of replacing discrete step protocols with continuous sweep protocols is that the latter can give a better resolution for parameter setting responses in less time, however, it is not sure if or when this advantage outweighs the possible loss in accuracy and the results will depend on the sweep duration. Evaluating the effects of parameter settings between the actual steps of a discrete step protocol requires interpolation, whereas with a continuous sweep protocol all parameter settings within the sweep range can be actually tested.

While this study demonstrates the promising potential for continuous sweep protocols in studying human-robot interaction, we acknowledge that there remains significant work to understand how different protocol conditions (e.g. rate of change of robot parameters, study duration, uni- versus bidirectional sweeping, etc.) impact the wearer response (e.g. physiological dynamics and drift and adaptation effects). However, in this study, we have shown that using continuous sweep protocols for examining changes in kinematic or kinetic objectives has potential for improving over discrete step protocols. This could be useful for certain applications where metabolic rate is not the prime objective or participants have limited walking ability.

## Additional files

Additional file 1: Choice of curve fitting order for fitting metabolic rate versus peak exosuit ankle moment. (PDF 97 kb)

Additional file 2: Simulation of physiological dynamics. (PDF 71 kb)

Additional file 3: Inter-stride variability [36]. (PDF 734 kb)

Additional file 4: Biomechanical delay analysis in *Discrete* condition data. (PDF 49 kb)

Additional file 5: Change in metabolic rate plotted against exosuit ankle peak moment plus minus 95% confidence interval. (PDF 59 kb)
