# Supplementary material for: Continuous sweep versus discrete step protocols for studying effects of wearable robot assistance magnitude
Source: J Neuroeng Rehabil. 2017 Jul 12;14:72. doi: 10.1186/s12984-017-0278-2 (PMC5506663; doi:10.1186/s12984-017-0278-2)
Supplement: Supplementary file 1 — Choice of curve fitting order for fitting metabolic rate versus peak exosuit ankle moment. (PDF 97 kb) [file 12984_2017_278_MOESM1_ESM.pdf]

Additional file 1:

Choice of curve fitting order for fitting metabolic rate versus peak exosuit ankle moment

We evaluated mixed model-anova of change in metabolic rate versus peak exosuit ankle force with different polynomial orders descending from third order to linear (1 to 3). Using third order fitting most coefficients for all the conditions were not significant, suggesting that using third order would lead to overfitting. For *Continuous-up* and *Continuous-down* all terms except the intercept were significant for second order fits and *Discrete* also had a significant second order coefficient for change in metabolic rate versus peak exosuit ankle force, therefore second order fitting was selected for the analyses in the present manuscript.

Third order polynomial fitting for metabolic rate versus peak exosuit ankle force (1)

Discrete curve fit (With x = Peak exosuit ankle moment (N kg^{-1}))

Change in metabolic rate (W kg^{-1}) =	-0.0017159	-0.49741 x	-0.77514 x^2	-1.104 x^3
P- values =	0.9873	0.7062	0.8617	0.7831

Continuous-up curve fit (With x = Peak exosuit ankle moment (N kg^{-1}))

Change in metabolic rate (W kg^{-1}) =	0.16425	-0.58093 x	-1.2816 x^2	+0.46482 x^3
P- values =	0.0346	0.1043	0.2648	0.6549

Continuous-down curve fit (With x = Peak exosuit ankle moment (N m kg^{-1}))

Change in metabolic rate (W kg^{-1}) =	0.15818	-1.5598 x	-1.4173 x^2	+ 2.1353 x^3
P- values =	0.1326	0.0001	0.2711	0.069

Second order polynomial fitting metabolic rate versus peak exosuit ankle force (2)

Discrete curve fit (With x = Peak exosuit ankle moment (N kg^{-1}))

Change in metabolic rate (W kg^{-1}) =	-0.013657	-0.1705 x	-1.9821 x^2
P- values =	0.8888	0.7623	0.0124

Continuous-up curve fit (With x = Peak exosuit ankle moment (N kg^{-1}))

Change in metabolic rate (W kg^{-1}) =	0.17254	-0.72616 x	-0.77597 x^2
P- values =	0.0222	0.0000	0.0001

Continuous-down curve fit (With x = Peak exosuit ankle moment (N kg^{-1}))

Change in metabolic rate (W kg^{-1}) =	0.19729	-2.2172 x	+ 0.88252 x^2
P- values =	0.0555	0.0000	0.0001

Linear fitting metabolic rate versus peak exosuit ankle force (3)

Discrete curve fit (With x = Peak exosuit ankle moment (N kg^{-1}))

Change in metabolic rate (W kg^{-1}) =	0.12235	-1.597 x
P- values =	0.1654	0.0000

Continuous-up curve fit (With x = Peak exosuit ankle moment (N kg^{-1}))

Change in metabolic rate (W kg^{-1}) =	0.23653	-1.2791 x
P-values =	0.0013	0.0000

Continuous-down curve fit (With x = Peak exosuit ankle moment (N kg^{-1}))

Change in metabolic rate (W kg^{-1}) =	0.12907	-1.6131 x
P-values =	0.2097	0.0000
