# Supplementary material for: Continuous sweep versus discrete step protocols for studying effects of wearable robot assistance magnitude
Source: J Neuroeng Rehabil. 2017 Jul 12;14:72. doi: 10.1186/s12984-017-0278-2 (PMC5506663; doi:10.1186/s12984-017-0278-2)
Supplement: Supplementary file 2 — Simulation of physiological dynamics. (PDF 71 kb) [file 12984_2017_278_MOESM2_ESM.pdf]

Additional file 2: Simulation of physiological dynamics

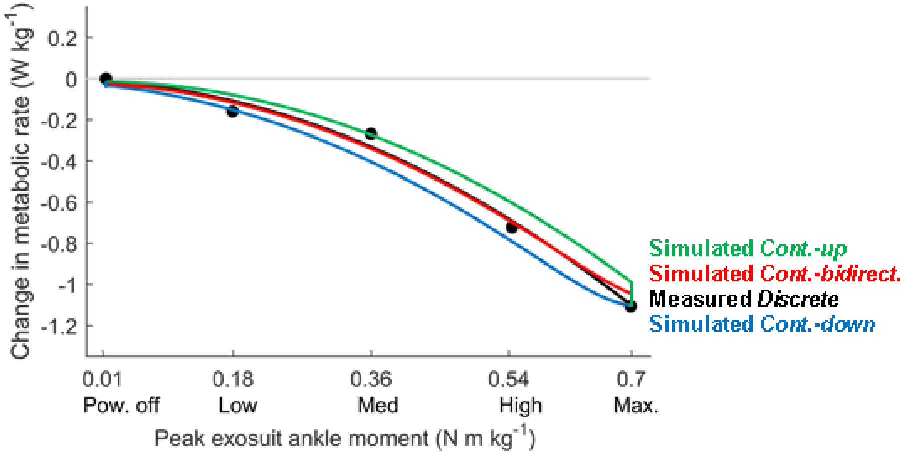

Simulated result for *Continuous-up* and *Continuous-down* calculated based off measured mean result from *Discrete* and assuming physiological dynamics characterized by exponential delay with a time constant of 42 s. The simulated results are calculated based on

$$\Delta E_{cost\ Continuous}(t) = (1 - (dt / \tau)) \cdot \Delta E_{cost\ Continuous}(t\text{-}1) + (dt / \tau) \cdot \Delta E_{cost\ Discrete}(t\text{-}1)$$

With $\tau = 42$ s and $\Delta E_{cost\ Continuous}(t_0) = \Delta E_{cost\ Discrete}(t_0)$

Black, green, blue and red line respectively represent population average second order polynomial curve fits for *Discrete*, simulation of *Continuous-up*, simulation of *Continuous-down* and the average of simulation of *Continuous-up* and simulation of *Continuous-down*. From this figure, it can be hypothesized that the effect of delay is small at low peak moments because changes in peak moment only lead to small changes in metabolic rate. It can also be seen that the average from simulation of *Continuous-up* and simulation *Continuous-down* will not correctly predict metabolic rate at *Max*. because of initialization effects.
