# Supplementary material for: Continuous sweep versus discrete step protocols for studying effects of wearable robot assistance magnitude
Source: J Neuroeng Rehabil. 2017 Jul 12;14:72. doi: 10.1186/s12984-017-0278-2 (PMC5506663; doi:10.1186/s12984-017-0278-2)
Supplement: Supplementary file 3 — Inter-stride variability [36]. (PDF 734 kb) [file 12984_2017_278_MOESM3_ESM.pdf]

Additional file 3: Inter-stride variability

Table: Stride-to-stride variability in kinematic parameters. Inter-stride-standard deviation over a 30-stride moving window after de-trending using the first-difference method [36] for peak dorsiflexion angle, peak plantarflexion angle and peak hip extension angle.

	**Curve fit evaluated at Med. peak force (s.e.m.)**			
	Cont.-up	*Discrete*	*Cont.-bidirect.*	*Cont.-down*
Peak dorsiflexion angle detrended stride-to-stride standard deviation (deg)	1.06 (0.07)	0.98 (0.06)	1.09 (0.08)	1.12 (0.09)
Peak plantarflexion angle detrended stride-to-stride standard deviation (deg)	1.74 (0.16)	1.78 (0.22)	1.77 (0.17)	1.80 (0.18)
Peak hip extension angle detrended stride-to-stride standard deviation (deg)	0.75 (0.06)	0.68 (0.07)	0.73 (0.06)	0.71 (0.07)

To evaluate variability in the different parameter study conditions we calculated standard deviations over a thirty-stride moving window on kinematic parameters. However, due to the slope of the increase or decrease in peak exosuit ankle moment in *Continuous-up* and *Continuous-down* a higher thirty-stride standard deviation is automatically expected in *Continuous-up*, *Continuous-down* and *Continuous-bidirectional*. To analyze differences in stride-to-stride variability independent from the slope of the peak moment increase or decrease we detrended the data first using the first-difference method similar to [36]. Results show that when the slope of the increase or decrease in peak moment is taken into account by detrending there are no significant differences in stride-to-stride variability in kinematic parameters between conditions. This absence of increased variability in *Continuous-bidirectional*, *Continuous-up* and *Continuous-down* suggests that participants were not perturbed by the slow linear increase or decrease peak moment. This echoes results from a recent split-belt treadmill study where it was assumed that participants can easily track treadmill speed changes when they are presented in a gradual way as in *Continuous-up* and *Continuous-down* [33].
