# Supplementary material for: Continuous sweep versus discrete step protocols for studying effects of wearable robot assistance magnitude
Source: J Neuroeng Rehabil. 2017 Jul 12;14:72. doi: 10.1186/s12984-017-0278-2 (PMC5506663; doi:10.1186/s12984-017-0278-2)
Supplement: Supplementary file 4 — Biomechanical delay analysis in Discrete condition data. (PDF 49 kb) [file 12984_2017_278_MOESM4_ESM.pdf]

Additional file 4: Biomechanical delay analysis in *Discrete* condition data

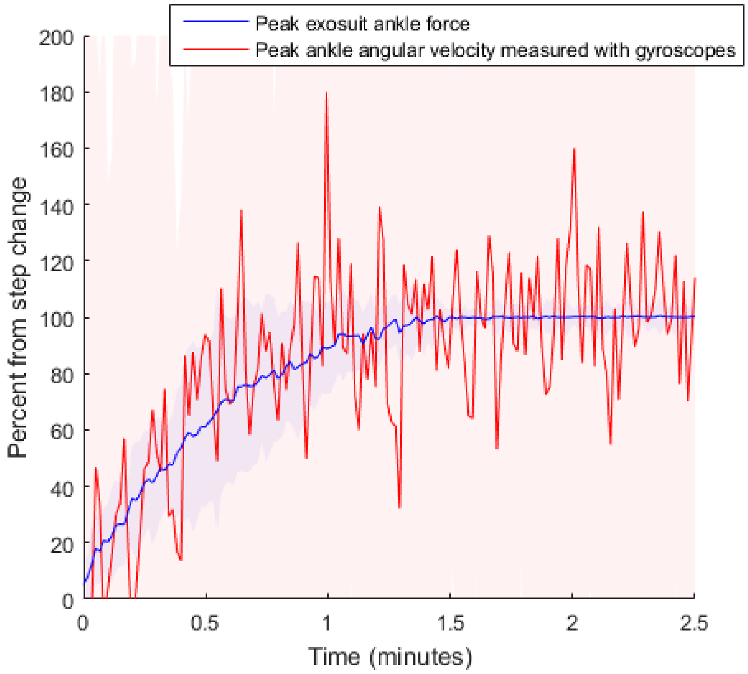

We analyzed biomechanical response delay on a chosen kinematical data. We selected peak dorsiflexion angle because this parameter is significantly affected by the exosuit. Blue line shows median of change in peak force over the first 2.5 minutes of every discrete step change. Red line is median of change in peak dorsiflexion velocity measured by the gyroscopes over the first 2.5 minutes of every step change. Blue and red shaded areas represent interquartile distance. In order to allow averaging changes in peak force and peak dorsiflexion velocity from discrete step changes with different magnitudes and directions (depending on the randomization of the protocol) these magnitude changes were normalized such that 0% is the average value from the last minute before the discrete step change and 100% is the average value from the last minute after the discrete step change.
