# Supplementary material for: Continuous sweep versus discrete step protocols for studying effects of wearable robot assistance magnitude
Source: J Neuroeng Rehabil. 2017 Jul 12;14:72. doi: 10.1186/s12984-017-0278-2 (PMC5506663; doi:10.1186/s12984-017-0278-2)
Supplement: Supplementary file 5 — Change in metabolic rate plotted against exosuit ankle peak moment plus minus 95% confidence interval. (PDF 59 kb) [file 12984_2017_278_MOESM5_ESM.pdf]

Additional file 5: Change in metabolic rate plotted against exosuit ankle peak moment plus minus 95% confidence interval.

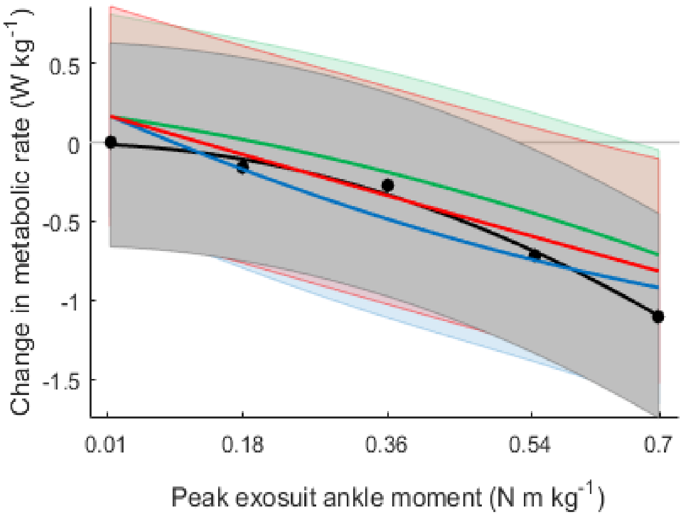

Change in metabolic rate plotted against exosuit ankle peak moment plus minus 95% confidence interval. Black, green, blue and red line respectively represent population average second order polynomial curve fits for *Discrete*, *Continuous-up*, *Continuous-down* and the average of *Continuous-up* and *Continuous-down* called *Continuous-bidirectional*. Shaded areas represent 95% confidence interval error of *Discrete*, *Continuous-up* and *Continuous-down* (for *Continuous-up* standard error is only plotted in the positive direction and for *Continuous-down* it is only plotted in the negative direction).
